# Regulation of Toll-like receptor 1 and -2 in neonatal mice brains after hypoxia-ischemia

**DOI:** 10.1186/1742-2094-8-45

**Published:** 2011-05-10

**Authors:** Linnea Stridh, Peter LP Smith, Andrew S Naylor, Xiaoyang Wang, Carina Mallard

**Affiliations:** 1 Institute of Neuroscience and Physiology, Sahlgrenska Academy, University of Gothenburg, Gothenburg, Sweden; 2 Center of Brain Research and Rehabilitation, Institute of Neuroscience and Physiology, University of Gothenburg, Gothenburg, Sweden; 3 Department of Pediatrics, the Third Affiliated Hospital of Zhengzhou University, 450052 Zhengzhou, China

## Abstract

**Background:**

Hypoxic-ischemic (HI) brain injury remains a major problem in newborns, resulting in increased risk of neurological disorders. Neonatal HI triggers a broad inflammatory reaction in the brain, including activation of the innate immune system. Toll-like receptors (TLRs), which are key components of the innate immune system, are believed to play a role in adult cerebral ischemic injury. The expression of TLRs in the neonatal brain and their regulation after HI is unknown.

**Methods:**

Wild type C57BL/6, TLR 1 knockout (KO) and TLR 2 KO mice were subjected to HI at postnatal day 9 and sacrificed 30 min, 6 h, 24 h or 5 days after HI. TLR mRNA expression was determined by RT-qPCR and protein and cell type localisation by immunohistochemistry (IHC). To evaluate brain injury, infarct volume was measured in the injured hemisphere.

**Results:**

mRNA expression was detected for all investigated TLRs (TLR1-9), both in normal and HI exposed brains. After HI, TLR-1 was down-regulated at 30 min and up-regulated at 6 h and 24 h. TLR-2 was up-regulated at 6 h and 24 h, and TLR-7 at 24 h. Both TLR-5 and TLR-8 were down-regulated at 24 h and 30 min respectively. IHC showed an increase of TLR-1 in neurons in the ipsilateral hemisphere after HI. TLR-2 was constitutively expressed in astrocytes and in a population of neurons in the paraventricular nucleus in the hypothalamus. No changes in expression were detected following HI. Following HI, TLR-2 KO mice, but not TLR-1 KO, showed a decreased infarct volume compared to wild type (p = 0.0051).

**Conclusions:**

This study demonstrates that TLRs are regulated after HI in the neonatal brain. TLR-1 protein was up-regulated in injured areas of the brain but TLR-1 KO animals were not protected from HI. In contrast, TLR-2 was constitutively expressed in the brain and TLR-2 deficiency reduced HI injury. These data suggest that TLR-2, but not TLR-1, plays a role in neonatal HI brain injury.

## Background

Perinatal brain injury remains a major clinically acute and chronic problem resulting in increased risk of neurological disorders such as cerebral palsy and epilepsy. Although the precise aetiology of brain injury in the newborn is sometimes unclear, hypoxia-ischemia (HI) is well accepted as a contributing factor [1]. Both infection and intrapartum asphyxia is associated with inflammation in the brain [2] and there are increased levels of cytokines in the cerebral spinal fluid in term infants that have suffered birth asphyxia [3]. Experimental studies demonstrate that neonatal HI triggers a broad inflammatory reaction in the brain which includes activation of the innate immune system [4] and several studies in neonatal animals have shown that inhibition of pro-inflammatory mediators are neuroprotective [5-8].

Toll-like receptors (TLRs) are key components of the innate immune system, which recognise a wide variety of pathogen-associated molecular patterns (PAMPs), such as lipopolysaccharide, bacterial DNA, and double-stranded RNA (for reviews see [9-11]). The TLR family consists of 13 members and TLR 1-9 are expressed in both mice and humans. Upon activation, each toll-like receptor, except TLR-3, signals through the myeloid differentiation primary response gene 88 (MyD88)-dependent pathway. MyD88 is an adaptor protein, which upon recruitment to the activated receptor initiates a signalling cascade leading to activation of different transcription factors, e.g. nuclear factor κB (NFκB) and activator protein-1 (AP1). This activation then gives rise to a generation of pro-inflammatory cytokines such as interleukin (IL)-6 and tumour necrosis factor-α (TNF-α). In contrast, TLR-3 signals through the MyD88-independent pathway, initiated by the Toll/IL-1R domain containing adaptor inducing IFN-β (TRIF) molecule. Recruitment of TRIF leads to the activation of the transcription factor interferon regulatory factor (IRF) -3 and -7 and the generation of antiviral molecules such as interferon (IFN)-β.

In addition to their role in bacterial and viral infections, recent studies have shown that TLRs also recognise and are activated by endogenous molecules associated with damaged cells and tissues [12-14]. For example, Lehnardt *et al. *demonstrated that the intracellular chaperone heat shock protein 60 released from dying cells in the central nervous system activates microglia through a TLR-4- and MyD88-dependent pathway [13]. In addition, Karikó *et al. *revealed that RNA released from or associated with necrotic cells stimulated TLR-3 and induced an immune response [12]. Furthermore, growing evidence suggests that TLRs play a role in ischemic brain damage. In adult studies, TLR-4 has been found to be up-regulated after cerebral ischemia reperfusion [15] and mice lacking TLR-2 or TLR-4 are less susceptible to hypoxic/ischemic brain damage [16-19].

Most TLRs are constitutively expressed in the adult brain [20,21]. However, the expression of TLRs in the neonatal brain and how they are regulated after HI is unknown. Here we examine the expression of TLR 1-9 in the neonatal brain, both in control animals and after HI using a well-established animal model of perinatal brain damage [6,22]. Furthermore, we examined the functional role of TLR-1 and TLR-2 after HI.

## Methods

### Animals

C57BL/6 mice were purchased from Charles River (Sulzfeld, Germany). TLR-1 knock out (KO) mice were purchased from Oriental BioService, Inc (Tokyo, Japan) and TLR-2 KO mice (B6.129-Tlr2tm1Kir/J) were bought from the Jackson Laboratory (USA). All KO mice were on the C57BL/6 background. Mice were housed in a 12 h light-dark cycle and bred at Experimental Biomedicine (Gothenburg University, Gothenburg, Sweden) and were provided with a standard laboratory chow diet (B&K, Solna, Sweden) and drinking water *ad libitum*. All animal experiments were approved by the Ethical Committee of Gothenburg (No.277-07 and 374-09).

### Induction of hypoxia-ischemia in neonatal mice

At postnatal day (PND) 9, mice were anesthetised with isoflurane (3.0% for induction and 1.0-1.5% for maintenance) in a mixture of nitrous oxide and oxygen (1:1). The left common carotid artery was ligated with prolene sutures (the whole procedure was less than 5 min). Mice were returned to the cage and allowed to recover for 1 h and then placed in an incubator circulated with a humidified gas mixture (10.00 ± 0.01% oxygen in nitrogen) at 36°C for 50 min. After hypoxia, the pups were returned to their dam until sacrifice. Control animals received no surgery and were not subjected to HI. Two wild type and one TLR-1 KO mice died during the HI procedure.

### Tissue collection and processing

For mRNA expression analysis, wild type pups were deeply anesthetized and intracardially perfused with saline at 30 min (n = 5), 6 h (n = 5) and 24 h (n = 5) after HI. Brains were rapidly dissected out, divided into ipsi- and contralateral hemispheres, snap frozen and then stored at -80°C until analysis. Brain tissue was homogenised with Qiasol lysis reagent homogeniser (Qiagen, Solna, Sweden) and total RNA was extracted using RNeasy Lipid Tissue Mini Kit (Qiagen, Solna, Sweden) according to the manufacturer's instructions. RNA was measured in a spectrophotometer at 260-nm absorbance.

For immunohistochemical analysis, animals were deeply anesthetised and intracardially perfused with saline and 5% buffered formaldehyde (Histofix; Histolab, Sweden) at 30 min, 6 h, 24 h and 5 days after HI. Brains were rapidly removed and immersion fixed in 5% formaldehyde for 24 h. Brains were then kept in a 30% sucrose solution until they were cut or put through dehydration with graded ethanol and xylene and embedded in paraffin. Coronal sectioning (25 μm/section) was performed on a sliding microtome (Leica SM2000R, Leica Microsystems, Sweden), and sections were stored in tissue cryoprotectant solution (25% ethylene glycol, 25% glycerol and 0.1 M phosphate buffer) at -20°C. For detection of infarction volume paraffin embedded tissue was used. Paraffin embedded brains were cut coronally (10 μm/section) on a rotating microtome (Leica RM2165, Leica Microsystems, Sweden).

### TLR signalling pathway RT2-PCR-*Profiler *PCR Array

cDNA-synthesis was performed by using the RT^2 ^First Strand Kit (SABiosciences, Frederick, MD, USA) following the manufacturer's instructions. The mouse TLR signalling pathway RT^2^-PCR-*Profiler *PCR Array (SABiosciences, Frederick, MD, USA) was carried out according to manufacturer's instructions using the LightCycler 480 system (Roche, Bromma, Sweden). The raw data obtained from the Lightcycler 480 software was uploaded into GEarray Analyzer software (SABiosciences, Frederick, MD, USA) for analysis.

### Reverse transcription-quantitative PCR

To confirm the RT^2^-PCR-*Profiler *PCR Array results, TLR-1 and -2 mRNA expressions were determined by reverse transcription-quantitative PCR (RT-qPCR). Superscript RNase H- reverse transcriptase kit (Invitrogen, CA, USA) random hexamer primers and dNTP (Roche Molecular Biochemicals, IN, USA) were used to synthesise first strand cDNA as previously described [23]. Each PCR (20 μl) contained 2 μl cDNA diluted 1:8, 10 μl Quanti Fast SYBR Green PCR Master Mix (Qiagen, Sweden) and 2 μl PCR primer. The following primers were used: TLR-1 QuantiTech Primer Assay (QT00157430), TLR-2 QuantiTech Primer Assay (QT00129752) and Hprt-1 QuantiTech Primer Assay (QT00166768), all from Qiagen, Sweden. The amplification protocol comprised an initial 5 min denaturation at 95°C, followed by 40 cycles of denaturation for 10 sec at 95°C and annealing/extension for 30 sec at 60°C on a LightCycler 480 (Roche, Sweden). Melting curve analysis was performed to ensure that only one PCR product was obtained. For quantification and for estimation amplification efficiency, a standard curve was generated using increasing concentrations of cDNA. The amplification transcripts were quantified with the relative standard curve and normalised against the reference gene hypoxanthine guanine phosphoribosyltransferase-1(Hprt-1).

### Immunohistochemistry

Immunohistochemical staining was performed on free-floating 25 μm sections pretreated with 0.6% H_2_O_2 _in Tris-buffered saline (TBS; 0.15 M NaCl and 0.1 M Tris-HCl, pH 7.5) for 30 min to block endogenous peroxidase activity. Nonspecific binding was blocked for 30 min in blocking solution (3% goat serum and 0.1% Triton-X 100 in TBS). After rinsing, sections were incubated with primary antibody (TLR-1, IMG-5012, 1:500, TLR-2, IMG-526, 1:100, Imgenex, CA, USA) in blocking solution at 4°C for 48 h. The tissue sections were then incubated for 1 h with biotinylated goat anti-rabbit IgG secondary antibody (1:500, Vector Laboratories, CA, USA) in blocking solution and then rinsed in TBS. Visualisation was performed using Vectastain ABC Elite (Vector Laboratories, CA, USA) with 0.5 mg/ml 3, 3'-diaminobenzidine (DAB) enhanced with 0,01% H_2_O_2 _and 0,04% NiCl (all from Sigma-Aldrich, Sweden). Sections were analysed on an Olympus BX60 fluorescence microscope equipped with an Olympus DP50 cooled digital camera.

For paraffin sections, antigen recovery was performed by boiling the sections in 10 mM sodium citrate buffer (pH 6.0) for 10 min. Nonspecific binding was blocked for 30 min in blocking solution (1% horse serum, 3% bovine serum albumin, 0.1% NaN_3 _in phosphate buffered saline (PBS)). Sections were incubated in primary antibody against microtubule-associated protein-2 (MAP-2; clone HM-2, 1:1000; Sigma-Aldrich) at 4°C overnight, followed by biotinylated horse anti-mouse secondary anti body (1:250, Vector Laboratories) for 60 min at room temperature. Visualization was performed using Vectastain ABC Elite with 0.5 mg/ml 3,3_-_-diaminobenzidine enhanced with 15 mg/ml ammonium nickel sulfate, 2 mg/ml β-D-glucose, 0.4 mg/ml ammonium chloride, and 0.01 mg/ml β-glucose oxidase (all from Sigma-Aldrich).

To identify the cell type specific localisation of TLRs, multi-immunofluorescence staining was performed. Non-specific binding was blocked for 30 min in blocking solution (3% donkey serum and 0.1% Triton-X 100 in TBS). TLR antibodies were then incubated simultaneously with antibodies against specific markers for neurons (NeuN 1:1000, MAB377, or alexa 488 conjugated NeuN 1:1000, MAB377X, Chemicon International, USA, and HuC/D 1:500, A21271, Molecular Probes, Netherlands), oligodendrocytes (Olig2 1:1000, AF2418, R&D systems, UK), microglia (Iba-1 1:1000, ab5076, Abcam, UK), and astrocytes (GFAP 1:1000, PCK-591P, Covance, USA) diluted in blocking solution for 48 h at 4°C. Samples stained for HuC/D were subjected to sodium citrate antigen retrieval (10 mM NaCi, pH6 for 30 min at 80°C) prior to blocking. Immunoreactivity was visualised via appropriate combinations of the following secondary antibodies: donkey anti-chicken DyLight 549 (1:1000, Jackson ImmunoResearch, USA), donkey anti-mouse Alexa 488, donkey anti rabbit Alexa 594, donkey anti-goat Alexa 647, and donkey anti-rabbit Alexa 488 (1:1000, all from Molecular probes, Netherlands). Multi-channel confocal images were captured with a Leica TCS SP2 confocal system (Leica, Heidelberg, Germany) with channel settings appropriate to the fluorophores present. Sequentially scanned, grey scale Z-stacks were pseudocolored and processed in ImageJ (version 1.42u; National Institutes of Health, Bethesda, MD, http://rsb.info.nih.gov/ij) before final processing in Adobe Photoshop (version 11.0.2; Adobe Systems Inc., San Jose, CA).

### Brain infarct evaluation

For brain infarct evaluation, coronal paraffin sections throughout the hippocampus were used. Every 40th section was stained for MAP-2 and analyzed under a Nikon Optiphot-2 microscope equipped with an Olympus DP50 cooled digital camera. Images were captured and processed using Micro Image version 4.0 (Olympus). Infarct area was assessed as the MAP-2 negative area in the ipsilateral hemisphere and total infarct volume was calculated according to the Cavalieri Principle using the following formula: V = ∑A × P × T, where V = total volume, ∑A = the sum of areas measured, P = the inverse of the sections sampling fraction, and T = the section thickness.

### Data analysis and Statistics

For normalisation of gene expression on the RT^2^-PCR-*Profiler *PCR Array, five housekeeping genes, β-glucuronidase, Hprt-1, heat shock protein 90 kDa alpha, glyceraldehyde-3-phosphate dehydrogenase, and β-actin, were used. The cycle threshold (C_T_) was determined for each sample and normalised to the average C_T _of the five housekeeping genes. A comparative C_T _method was used to calculate relative gene expression. Data are represented as fold change relative to control. The p-value was calculated using a student's t-test (two-tailed, equal variance).

Brain infarct data were analyzed with one way ANOVA followed by Dunnett's Multiple Comparison Test to compare total infarct volume between genotypes or infarct area at each brain level between groups (WT, TLR-1 KO and TLR-2 KO). Data are presented as mean ± SD and significance was set at p ≤ 0.05. All statistical analyses were performed using GraphPad Prism 4.0 (GraphPad Software).

## Results

### mRNA expression of TLRs in normal brain and after hypoxia-ischemia

mRNA expression was detected for all TLRs present on the array in the PND 9 mouse brain (Table 1). Following HI, TLR-1 mRNA was down-regulated at 30 min (fold change 0.52, p = 0.004) then up-regulated at 6 h (fold change 2.39, p = 0.00001) and 24 h (fold change 3.36, p = 0.00001); TLR-2 was up-regulated at 6 h (fold change 1.63, p = 0.005) and 24 h (fold change 2.27, p = 0.006) and TLR-7 was up-regulated at 24 h (fold change 2.16, p = 0.005). Solely down regulated gene expression after HI was found with TLR-5 at 24 h (fold change 0.57, p = 0.004) and TLR-8 at 30 min (fold change 0.39, p = 0.006). TLR-3, -4, -6 and -9 mRNA expression did not show any significant changes after HI.

**Table 1:** Fold change values for TLRs 30 min, 6 h and 24 h after HI

	**Fold change *(p-value)***		
	
	**30 min**	**6 h**	**24 h**
**TLR 1**	0.52 *(0.004)*	2.39 *(0.00001)*	3.36 *(0.00001)*
**TLR 2**	1.23 *(n.s)*	1.63 *(0.005)*	2.27 *(0.006)*
**TLR 3**	0.59 *(n.s)*	0.83 *(n.s)*	1.00 *(n.s)*
**TLR 4**	0.95 *(n.s)*	1.20 *(n.s)*	0.95 *(n.s)*
**TLR 5**	0.64 *(n.s)*	0.88 *(n.s)*	0.57 *(0.004)*
**TLR 6**	0.68 *(n.s)*	1.35 *(n.s)*	1.28 *(n.s)*
**TLR 7**	0.91 *(n.s)*	1.23 *(n.s)*	2.16 *(0.005)*
**TLR 8**	0.39 *(0.006)*	1.68 *(n.s)*	1.17 *(n.s)*
**TLR 9**	1.04 *(n.s)*	0.92 *(n.s)*	0.76 *(n.s)*

**Figure 1:**
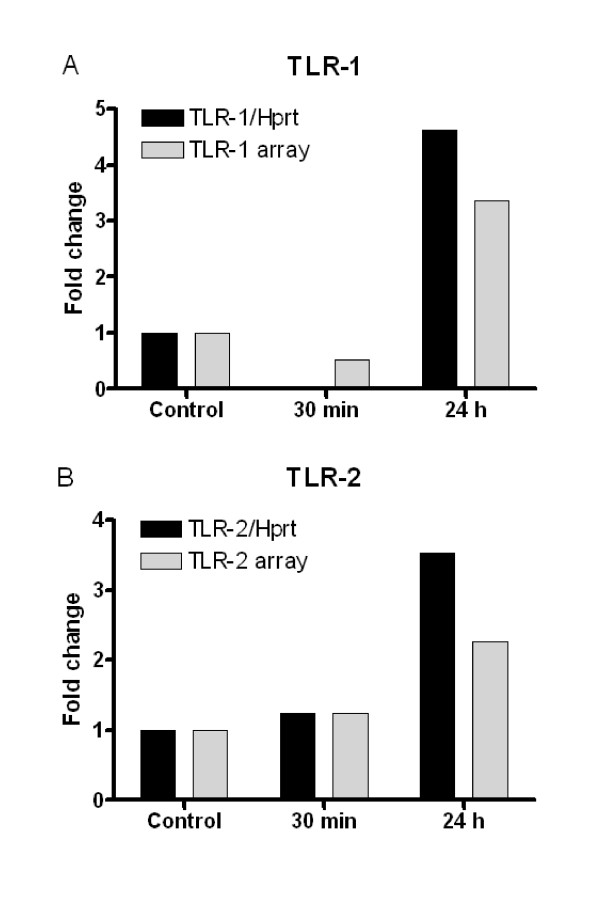
**Comparison of gene expression for TLR-1 and TLR-2 using TLR array and RT-qPCR**. Gene expression for TLR-1 (**A**) and TLR-2 (**B**) was confirmed with RT-qPCR (black bars) at 30 min and 24 h after HI and compared with TLR signalling pathway RT2-PCR-Profiler PCR Array (grey bars). RT-qPCR expression for TLR-1 and TLR-2 was calculated using Hprt-1 as housekeeping gene. Controls were not subjected to HI. n = 5 for 30 min, 24 h time points and controls.

To confirm the RT2-PCR-*Profiler *PCR Array results, mRNA expression levels for TLR1 and TLR2 were examined using RT-qPCR and normalised against the reference gene Hprt-1. TLR-1 and -2 were selected as they showed both mRNA up-and down-regulation on the array. A similar pattern of mRNA expression was observed for both TLR-1 and -2, when comparing the methods (Figure 1). On the array, TLR-1 showed a fold change of 0.52 at 30 min and 3.36 at 24 h after HI. When using RT-qPCR and the TLR-1 expression normalised against Hprt-1 the fold change was 0.02 at 30 min and 4.61 at 24 h (Figure 1A). For TLR-2, the array showed a fold change of 1.23 at 30 min and 2.27 at 24 h after HI. RT-qPCR for TLR-2 gave a fold change of 1.24 at 30 min and 3.53 at 24 h after HI when normalised against Hprt-1 (Figure 1B).

### Protein expression and distribution of TLR-1 and -2 in the brain after hypoxia-ischemia

To further study TLR regulation following injury, we performed immunohistochemical studies of the TLRs that displayed significant up-regulation of mRNA in response to HI, i.e. for TLR1 and 2. Immunohistochemical experiments performed using an antibody against TLR -7 were unsatisfactory and were not analysed further (data not shown).

**Figure 2:**
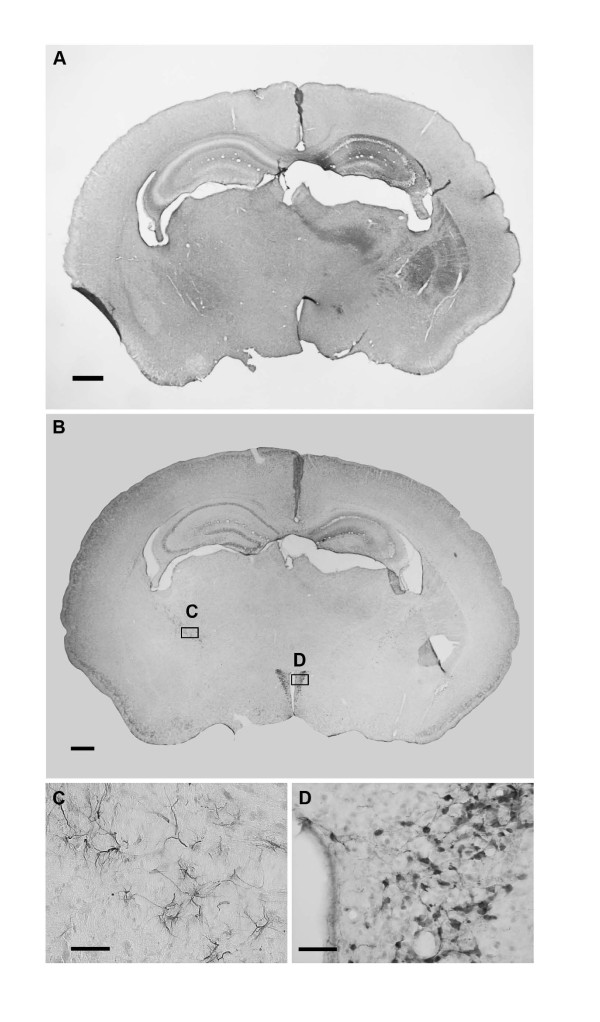
**TLR-1 and TLR-2 immunostaining 24 h after hypoxia-ischemia**. **A: **Representative picture of TLR-1 immunostaining 24 h after hypoxia-ischemia (HI). TLR-1 positive cells were found in the ipsilateral (damaged) side of the brain, primarily in hippocampus, striatum and thalamus. **B: **Representative picture of TLR-2 immunostaining 24 h after HI. TLR-2 positive cells were found in white matter e.g. stria terminalis and in the paraventricular nucleus of hypothalamus (PVN). **C: **High magnification of TLR-2 positive cells in stria terminalis representing the area of the C box in figure B. **D: **High magnification of TLR-2 positive cells in the PVN representing the area of the D box in figure B. Scale bars: A-B: 500 μm, C-D: 50 μm.

**Figure 3:**
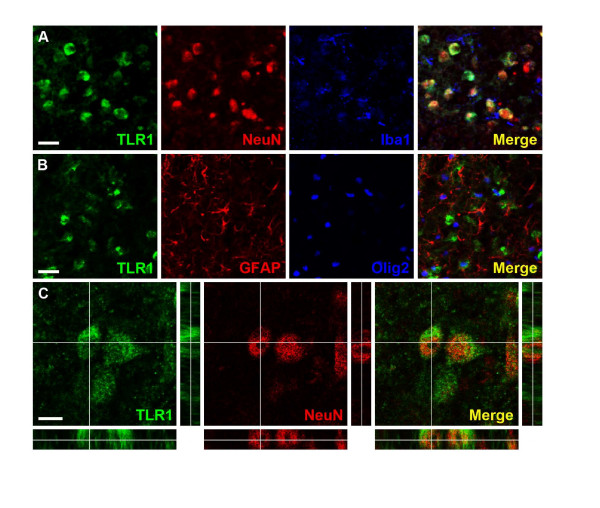
**Neurons express TLR-1 24 h after hypoxia-ischemia**. **A: **Triple immunofluorescence staining for TLR-1, NeuN and Iba-1; the merged picture displays expression of TLR-1 by NeuN positive neurons but not Iba-1 positive microglia. **B: **Triple immunofluorescence staining for TLR-1, GFAP and Olig2; The merged picture shows mutually exclusive expression of TLR-1, GFAP Olig2 and no co-localisation of all three markers. **C: **High magnification orthogonal views of cells stained with TLR-1 and NeuN; the merged picture shows membranous localisation of TLR-1 surrounding NeuN positive nuclei. All images were captured in the ipsilateral hippocampus. Scale bars: A-B: 25 μm C: 10 μm

No TLR-1 staining was detected in normal control brains or in the contralateral (non-damaged) hemisphere following HI. There was a marked increase of TLR-1 positive cells in the ipsilateral (damaged) hemisphere at 24 h, but not 30 min and 6 h after HI. TLR-1 positive staining was mainly observed in the hippocampus (Figure 2A) Double/triple-labelling experiments showed that TLR-1 was expressed in neurons (Figure 3A and 3C) but not in microglia, astrocytes or oligodendrocytes (Figure 3A and 3B).

**Figure 4:**
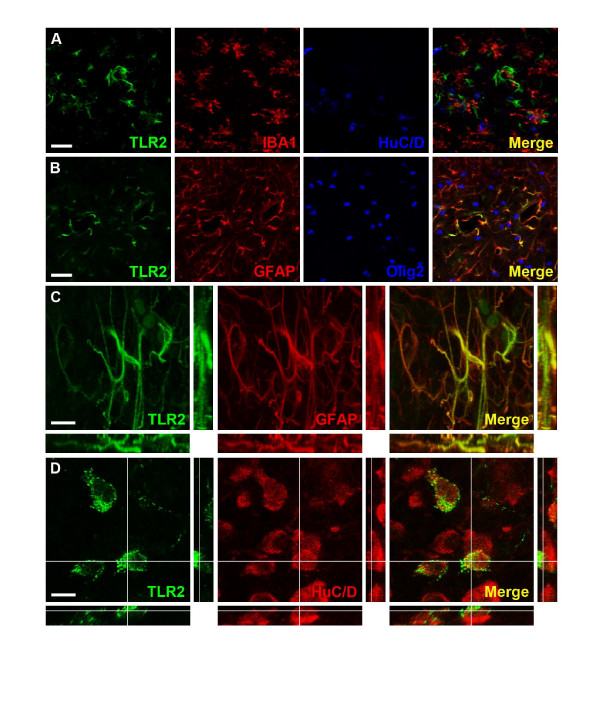
**TLR-2 is expressed in astrocytes and neurons**. **A: **Triple immunofluorescence staining for TLR-2, Iba-1 and HuC/D; the merged image indicates mutually exclusive expression of TLR-2, Iba-1 and HuC/D positive cells in the ipsilateral hippocampus. **B: **Triple immunofluorescence staining for TLR-2, GFAP and Olig2; the merged picture displays apparent co-expression of TLR-2 and GFAP. **C: **High magnification XYZ, XZ, YZ-average projections of TLR-2 and GFAP co-expressing cells; TLR-2 appears to be associated with cytoskeletal protein GFAP in astrocytes. Pictures A-C were captured in the ipsilateral hippocampus. **D: **High magnification orthogonal views of TLR-2 and HuC/D double stains in cells in the paraventricular nucleus of the hypothalamus (PVN); the merged picture shows punctate, membranous expression of TLR-2 on HuC/D positive neurons of the PVN. Scale bars: A-B: 25 μm C-D: 10 μm

TLR-2-immunoreactivity was observed mainly in the hippocampus, subcortical white matter and stria terminalis (Figure 2B and 2C). Co-staining experiments confirmed that TLR-2 positive cells in white matter areas were astrocytes but not neurons, microglia or oligodendrocytes (Figure 4A-C). A specific population of neuron-like cells expressing TLR-2 was found in the paraventricular nucleus of the hypothalamus (PVN, Figure 2B and 2D) and was confirmed to be neurons by co-staining experiments (Figure 4D). The immunohistochemical expression of TLR-2 did not change after HI.

### Brain infarct in wild type, TLR-1 and -2 KO animals after hypoxia-ischemia

**Figure 5:**
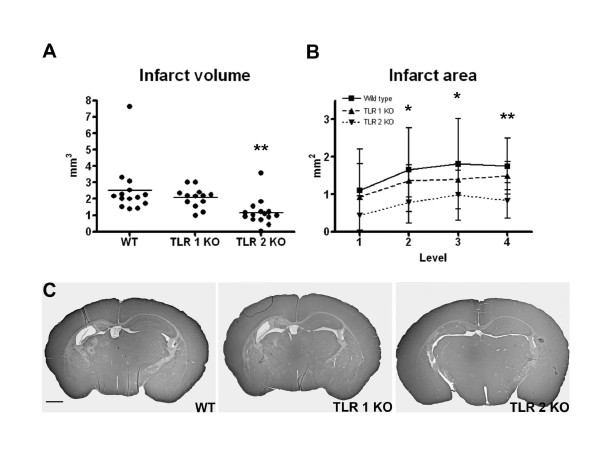
**Neuroprotection in TLR-2 KO mice after HI**. Neuropathological analysis at 5 days after HI showed a significant decrease in infarct volume (**A**) in TLR-2 KO mice (1.162 ± 0.791 mm^3^, n = 15) compared to WT (2.528 ± 1.570 mm^3^, n = 14, p = 0.0051). There was no protection in TLR-1 KO mice (2.068 ± 0.607 mm^3^, n = 13). The infarct area (**B**) was significantly attenuated in 3 out of 4 levels throughout hippocampus in TLR-2 KO mice compared to WT. **C: **Representative images of level 3 of the hippocampus for the different genotypes. Data is shown as mean ± SD. *≤ 0.05, **≤ 0.01. C: Scale bar: 1 mm.

To further evaluate the role of TLR-1 and TLR-2 in the neonatal brain after HI, we performed studies with TLR-1 and TLR-2 KO mice. Neuropathological analysis at 5 days after HI showed a significant decrease in infarct volume in TLR-2 KO mice (1.162 ± 0.791 mm^3^, p = 0.005, n = 15) compared to WT (2.528 ± 1.570 mm^3^, n = 14, Figure 5A). There was no protection in TLR-1 KO mice (2.068 ± 0.607 mm^3^, n = 13). The infarct area was significantly decreased in 3 out of 4 levels throughout hippocampus in TLR-2 KO mice compared to WT (Figure 5B). Figure 5C demonstrates representative images of level 3 of the hippocampus for the different genotypes.

## Discussion

We detected mRNA transcripts for all TLRs (TLR1-9) in the normal immature brain and regulation of mRNA expression for TLR-1, -2, -5, -7 and -8 after HI, with the largest up-regulation occurring for TLR-1 and TLR-2. Both TLR-1 and -2 immunoreactivity was also detected in the brain, however, only TLR-2 KO mice were protected from HI injury suggesting that TLR-2 may play an important role in neonatal HI.

In the present study we demonstrated the presence of TLR 1-9 mRNA in the normal neonatal brain under physiological conditions. Similarly, studies in human cerebral tissue have indicated that mRNA for most TLRs is expressed in numerous cell types [20]. In vitro studies also indicate a broad expression of TLR mRNA in numerous neural cell types [21,24-26], although the expression of neuronal TLR mRNA is more controversial [27]. Taken together, TLRs are expressed under basal conditions in both the immature and adult brain in numerous cell types and this may again suggest that they play essential roles in neurophysiology and pathology.

We showed that the mRNA expression for TLR-1, -2, -5, -7, and -8 was regulated after HI as shown on PCR array and confirmed by RT PCR. The regulation of TLR genes after neonatal HI is consistent with several studies in the adult brain. After 1 h of middle cerebral artery occlusion (MCAO), Lehnardt et al. found up-regulation of TLR-2 mRNA expression and Ziegler et al. found an induction of TLR-2, -4, and -9 mRNA after focal ischemia in mice [17,18]. Also, in a model of spinal cord injury, there was an increased mRNA expression of TLR-1, -2, -4, -5, and -7 [28]. This is to some extent similar to our results indicating an increase of TLR-1, -2, and -7 after HI. However, in contrast to studies in adult rodents, we found reduced mRNA expression of TLR-5 (24 h after HI) and TLR-8 (30 min after HI). TLR-3, -4, -6 and -9 were not significantly changed after neonatal HI, which suggests that the TLR response to cerebral ischemia differs in the adult compared to the developing brain.

We further investigated the protein distribution of the most regulated TLR genes following HI by immunohistochemical analysis. We found an increase of TLR-1, immunoreactivity in the ipsilateral hemisphere after HI, while TLR-2 was constitutively expressed in the neonatal brain and the expression did not change with HI. Thus, the pattern of protein expression after HI differed to some extent to the mRNA expression. Similarly, Mishra et al. have found differences between mRNA and protein expression and speculate that TLRs can be post-transcriptionally regulated [21].

In the HI model used in this study, brain injury is predominantly found in the hippocampus, striatum and thalamus [7]. Interestingly, TLR-1 showed increased immunoreactivity in neurons, but not other cell types in these brain regions. To our knowledge, there are no previous studies of TLR-1 expression after ischemic injury, either in the adult or developing brain. However, after induction of neurocysticercosis in adult mice, TLR-1 staining was found almost exclusively in infiltrating cells such as microglia/macrophages [21]. These differences in expression may be due to age-and/or injury- dependent factors. Other TLRs have been suggested to have adverse effects on brain development. Deficiency of TLR-3, which is predominantly expressed during embryonic life, increases proliferation of neural progenitor cells [29]. Ma et al. have shown that TLR-8 is expressed on neurons and axons during mouse brain development and that stimulation of this receptor leads to inhibition of neurite outgrowth and induction of apoptosis [30]. Taken together, these studies give support to the hypothesis that activation of certain TLRs can be harmful to the developing brain; however, based on the findings in the present study, the increased expression of TLR-1 in the ischemic brain does not appear to contribute to neonatal damage. In support, we have previously shown that mice deficient in the TLR-1 down-stream adaptor protein MyD88 are not protected from neonatal HI injury [31].

The immunohistochemical expression pattern of TLR-2 was different to that of TLR-1, with a constitutive expression both in white matter astrocytes and in a specific population of neurons in PVN. TLR-2 expression has previously been detected in microglia, neurons, ependymal cells and astrocytes, [21] and constitutive expression of TLR-2 has been demonstrated by in situ hybridisation specifically in stria terminalis, PVN, and in the supraoptic nucleus [32]. Thus, our results correspond well with previous reports. Existing studies have shown both a neuroprotective [33] and a damaging [17,18,26,34] role for TLR-2 in the injured brain. It has also been demonstrated that cerebral cortical neurons up-regulate TLR-2 in response to ischemia/reperfusion injury and that an increased number of apoptotic neurons are found in the murine dentate gyrus 24 h after stimulation with a TLR-2 agonist [26,34]. Other studies have found that TLR-2 is involved in adult hippocampal neurogenesis [35] and that after a spinal cord injury, TLR-2 is important for the regulation of inflammation and gliosis as well as the repair and functional recovery[28]. We have recently found that repeated stimulation of the TLR-2 receptor during early neonatal development by administration of the TLR-2 agonist Pam3CSK4 results in brain injury and this effect is blocked in TLR-2 deficient mice [36]. Thus these data in neonatal animals support the findings in the present study where TLR-2 KO animals demonstrated a decrease in infarct volume following HI. These results may seem contradictory to our previous results showing no neuroprotective effect in the TLR-2 adaptor protein MyD88. However, studies in adult animals have also shown a differential effect of MyD88 KO and TLR-2 KO following brain damage [37].

We found a specific population of TLR-2 positive neurons in the PVN. Inflammatory stimuli, such as LPS, are known to activate secretory neurons in PVN, a neuronal subpopulation that is important for homeostatic control [38]. Specifically, TLR-4 and MyD88 have been implicated in circadian responses and anorexia respectively [39,40]. The function of the TLR-2 immunoreactive neurons in PVN is unknown but we speculate that they may participate in the neuroendocrine response of the hypothalamus and may act as sensors for incoming inflammatory signals.

## Conclusions

This study demonstrates that a number of TLRs are regulated after HI in the neonatal brain, both on the mRNA and protein level. TLR-1 protein expression was found to be up-regulated in damaged areas of the brain, however based on the studies in TLR-1 deficient mice, did not appear to be involved in the injurious processes causing ischemic injury. In contrast, TLR-2 protein expression was constitutively expressed and did not change after HI. On the other hand TLR-2 deficiency did protect the immature brain from HI damage, which may indicate that TLR-2 plays a role in neonatal ischemic injury.

## Competing interests

The authors declare that they have no competing interests.

## Authors' contributions

CM, LS and XW conceived and designed the study; LS, PS and XW performed and analysed the experiments. LS, CM, PS and AN drafted the manuscript. All authors have read and approved the final version of the manuscript.
